# Transportation of Berberine into HepG2, HeLa and SY5Y Cells: A Correlation to Its Anti-Cancer Effect

**DOI:** 10.1371/journal.pone.0112937

**Published:** 2014-11-17

**Authors:** Yu-Nong Pang, Yin-Wen Liang, Tian-Shi Feng, Shuang Zhao, Hao Wu, Yu-Shuang Chai, Fan Lei, Yi Ding, Dong-Ming Xing, Li-Jun Du

**Affiliations:** 1 MOE Key Laboratory of Protein Sciences, Laboratory of Molecular Pharmacology and Pharmaceutical Sciences, School of Life Sciences and School of Medicine, Tsinghua University, Beijing, China; 2 NGM Biopharmaceuticals, Inc., South San Francisco, California, United States of America; 3 Drug Discovery Facility, School of Life Sciences, Tsinghua University, Beijing, China; University of Crete, Greece

## Abstract

The anti-cancer activities of berberine (BBR) have been reported extensively in various cancer cell lines. However, the minimal inhibitory concentrations of BBR varied greatly among different cell lines and very few studies have been devoted to elucidate this aspect. In this study, we employed three cancer cell lines, HepG2, HeLa and SY5Y, to compare the transportation and distribution of BBR. HPLC results demonstrated that BBR was capable of penetrating all the cell lines whereas the cumulative concentrations were significantly different. HepG2 cells accumulated higher level of BBR for longer duration than the other two cell lines. Molecular docking studies revealed the BBR binding site on P-glycoprotein 1 (P-gp). In addition, we elucidated that BBR regulated P-gp at both mRNA and protein levels. BBR induced the transcription and translation of P-gp in HeLa and SY5Y cells, whereas BBR inhibited P-gp expression in HepG2 cells. Further study showed that BBR regulates P-gp expression depending on different mechanisms (or affected by different factors) in different cell lines. To summarize, our study has revealed several mechanistic aspects of BBR regulation on P-gp in different cancer cell lines and might shed some useful insights into the use of BBR in the anti-cancer drug development.

## Introduction

Berberine (BBR), an isoquinoline alkaloid, can be isolated from medicinal plants such as *Rhizoma coptidis*, *Scutellaria baicalensis* and *Phellodendron amurense*. Although it is mainly used to treat infectious diseases in traditional medical practice, BBR has recently been explored for lipid-lowering, anti-diabetes, immune modulation, protection of ischemic myocardium, anti-hypertension, anti-arrhythmia and anti-cancer purposes in several studies [1], [2].

The anti-cancer effect of BBR has been reported on many cancer cell lines, including PC12 cells [3], melanoma cells [4], breast cancer cells [5], human colon cancer cells [6], 3T3-L1 adipocytes [7], non-small cell human lung cancer cells [8], prostate cancer cells [9], liver cancer cells [10], cervical cancer cells [11], etc. It is reported that the minimal inhibitory concentration of BBR varied significantly among different cancer cell lines, from 10 nM (3.73 ng/ml) in colon carcinoma cell to 400 µM (149.2 µg/ml) in MHCC97-L cells, through an unknown mechanism [12], [13]. Previous studies also demonstrated that BBR, a class of alkaloid DNA intercalators, was imported via a cationic transporter. But the intracellular distribution of BBR is still unclear [14]. P-gp, the most extensively studied member of ATP binding cassette (ABC) membrane efflux transporters, has been identified as a major transporter responsible for the efflux of BBR. The inhibition of P-gp was found to increase the intracellular concentration of BBR [15]. Recent studies reported that BBR regulates P-gp at different extends in different cell lines [16]. However, regulation of P-gp at mRNA and protein levels by BBR has not been reported yet.

In this study we chose three widely used cancer cell lines, HepG2, HeLa and SY5Y to evaluate the intracellular transportation, distribution and efflux of BBR, aiming to understand the mechanism underlying the different responses to BBR among different cell lines.

## Materials and Methods

### Cell culture and cytotoxicity assay

HepG2 cells were provided by Dr. Li Cao, Institute of Medicinal Plant Development (IMPLAD). HeLa cells were purchased from American Type Culture Collection (ATCC) (Rockvill, Maryland, USA). Both cell lines were cultured in DMEM supplemented with 10% FBS, penicillin (100 U/ml) and streptomycin (100 mg/ml). SY5Y cells were provided by the Cell Bank of the Institute of Fundamental Medicine, Chinese Academy of Medical Sciences (Beijing, China), cultured in RPMI Medium 1640 supplemented with 10% FBS, penicillin (100 U/ml) and streptomycin (100 mg/ml). Cells were cultured at 37°C under 5% CO_2_ and 90% humidity. The IACUC (Institutional Animal Care and Use Committee) of Tsinghua University approved all protocols used in this study (Approval ID: 2013-DuLJBBR001).

The cytotoxicity of BBR in the three cell lines was evaluated by an MTT (3-(4,5-dimethy-lthiazol-2-yl)-2,5-diphenyltetrazolium bromide) assay. The MTT assay was performed according to the method described by Chai et al. [17].

### Confocal microscopy

Cells were treated with BBR (0.5 µg/ml in water) or water alone after reaching 70% confluence [14]. After drug treatment for 12 h, cells were used for microscopy imaging. The fluorescence of intracellular BBR was observed with excitation at 405 nm and emission at 520 nm. Images were taken under a Zeiss LSM Meta 710 Confocal Microscope equipped with argon and helium-neon lasers (Carl Zeiss, Germany) and further analyzed by Zen 2011 Software.

### High performance liquid chromatography (HPLC) assay

An HPLC system containing an Agilent 1260 HPLC system (1260 Quat Pump, 1260 DAD, 1260 ALS) and a C-18 HPLC column (150 mm×3.9 mm, 5 µm particle size silica, Waters, Ireland) was used to determine the intracellular concentration of BBR. A mobile phase consisting of acetonitrile/water (28/72, v/v) containing 0.5% triethylamine was pumped through the column at 0.8 ml/min. BBR was detected by UV absorbance at a wavelength of 347 nm [18]. Under these conditions, the retention time of BBR was 6.8 min. The quantitative linear range was 0–5000.0 ng/ml for BBR. Standard curves of BBR were drawn using weighted linear regression of peak area ratio values of the calibration standards. The correlation coefficient (R^2^) was 0.999.

### Liquid chromatography-mass spectrometer (LC/MS) assay

An Agilent 1200/6340 LC/MS was utilized to quantify intracellular BBR. Intracellular BBR was extracted using methanol and flowed through a C-18 HPLC column (150 mm×3.9 mm, 5 µm particle size silica, Waters, Ireland) at a speed of 0.2 mL/min at 25°C. The mobile phase consisted of solvent A (10 mM ammonium acetate, 0.1% methanoic acid in water and adjust pH to 3) and solvent B (acetonitrile) was used to equilibrate the column in the following gradient: 15% solvent B for 2 min, 30% solvent B for 11 min, 90% solvent B for 12 min and finally 15% solvent B for 5 min. Under these conditions, the retention time of BBR was 12.6 min. BBR was monitored at mass/charge ratio (m/z) of 336.0.

### Computer based docking study

The reported crystal structure of P-gp, downloaded from Protein Data Bank website (PDB code: 2YL2, http://www.rcsb.org/pdb/explore/explore.do?structureId=2YL2) was chosen as the docking template. The electron density data was acquired from the Electron Density Server web site (http://eds.bmc.uu.se/cgi-bin/eds/uusfs?pdbCode=2YL2). The structure files of the protein and BBR molecule were analyzed by AutoDockTools-1.5.4. The grid box included the whole transmembrane domain.

### Nucleotide acid manipulation and transformation

The construction of P-gp promoter fused GFP expression (P-gp promoter-GFP) was generated using general molecular technique. To replace the *SpeI* and *XbaI* fragment of the pEGFP-N1 vector, a 1 kb P-gp promoter was amplified by PCR from a reverse PCR of HepG2 cell [19]. Primers used were: 1Kb-AseI-F: 5′-TTTATTAATCTGCAGAAAAATTTCTCCTAG and 1Kb-BamHI-R: 5′-CGCGGATCCCTGCAGGGGCTTTCCT.

This P-gp promoter fused GFP construction was verified by sequencing and subjected to transfect into HepG2, HeLa and SY5Y cells by electroporation separately. Cells in serum-free medium containing 20 µg DNA were electroporated in an electroporator (Model ECM 630, BTX). The electroporation parameters set were 250 V voltage (low voltage model), 1575 Ω resistance and 1000 µF capacitance. Cells were pulsed twice with 1 min interval. After electroporation, cells were transferred into 10 ml dishes with 10% serum and recovered for 24 h followed by drug treatments [20].

### Quantitative RT-PCR

The mRNA levels were determined using SYBR Green based quantitative PCR kit (Tiangen, China). Total RNA was isolated by Trizol Total RNA Isolation kit (Tiangen, China). RNA sample of 50 ng was reverse transcribed using M-MLV First Strand cDNA Synthesis Kit (TransGen, China). The cDNA products were confirmed by DNA gel electrophoresis. Quantitative PCR was performed as described previously [21]. Primers used were 5′-GCTGGATGTTTCCGGTTTGG and 5′-TTCCGTGCTGTAGCTGTCAA for P-gp, 5′-CATGTACGTTGCTATCCAGGC and 5′-CTCCTTAATGTCACGCACGAT for *β*-actin. 5′-GCAGAAGAACGGCATCAAGG and 5′-CGGACTGGGTGCTCAGGTAG for GFP. The cycling conditions were: 94°C for 3 min; 40 cycles of 94°C for 10 sec, 60°C for 10 sec, 72°C for 20 sec; 72°C for 10 min and cooled to 4°C. Data were processed using the LC-480 II software program (Roche, USA).

### Western blot

The protein samples were prepared and used for Western blotting as described previously [22]. The protein concentration was determined using Bio-Rad protein assay reagent. Protein samples were resolved on 8% SDS-polyacrylamide gels and transferred to nitrocellulose membranes (Bio-Rad, USA). Membranes were blocked with 5% non-fat dry milk in TBST (TBS buffer containing 0.05% Tween-20) for 1 h at room temperature and incubated with the primary antibody diluted with 3% milk in TBST at 4°C overnight. The primary antibodies used were mouse monoclonal anti-P-gp (1∶500, Santa Cruz, Santa Cruz, CA), GFP (1∶3000, Abmart, Shanghai, China) and β-actin (1∶2000, Boster Biological Technology, Wuhan, China). The secondly antibody used was goat-anti-mouse HRP antibody (1∶1000, ZSGB-BIO, China), followed by chemiluminescence as described [21].

### Statistical analysis

All data were represented as mean ± S.D. and statistically analyzed using one-way analysis of variance (ANOVA). The F test was carried out using Excel Software for Office 2007 (Microsoft, U.S.). After the F test, the student’s *t*-test between two groups was performed. One-way ANOVA *t*-test was employed to analyze the differences between sets of data using Graph Pad Prism 5.0 software (Graph Pad, San Diego, U.S.). *P*<0.05 was considered statistically significant.

## Results

### Cellular uptake of BBR

**Figure 1:**
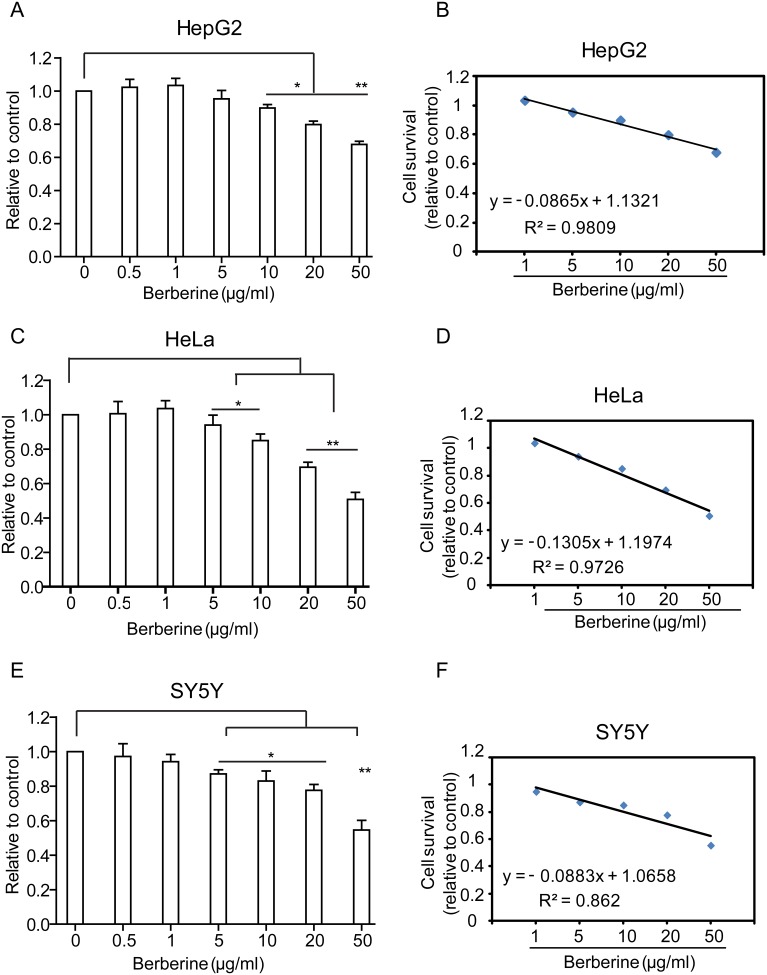
Cytotoxicity of berberine (BBR) in three cell lines. (A–F) The cytotoxicity and lethality of BBR using MTT assay. (A, B) HepG2, (C, D) HeLa. (E, F) SY5Y. *P<0.05, **P<0.01, ***P<0.005, statistical significance of BBR treated groups relative to control groups. Data are presented as mean ± S.D. from six independent experiments (n = 6).

**Figure 2:**
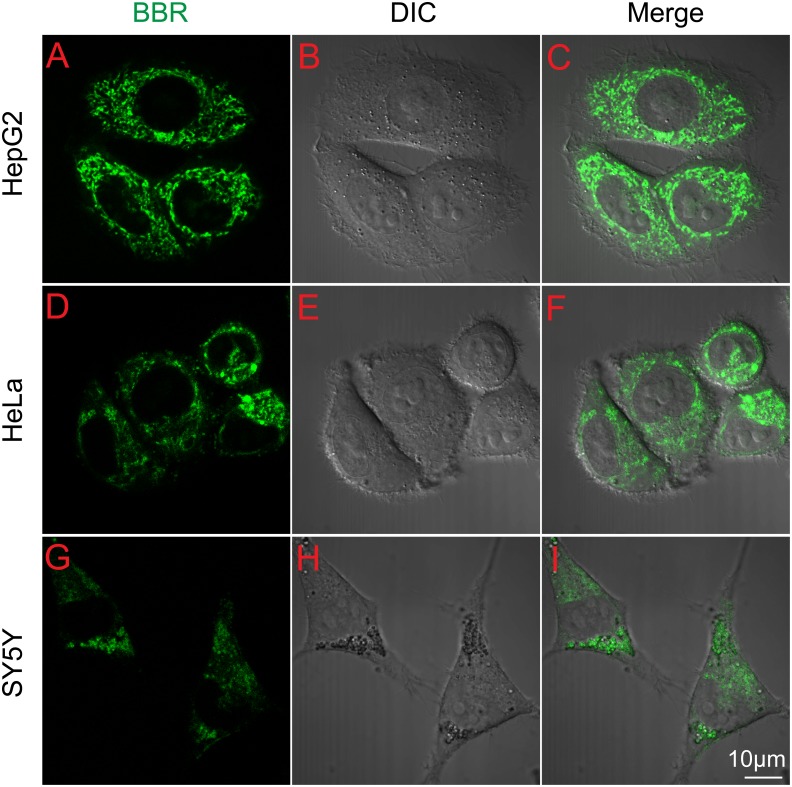
Transportation of BBR through the cells. (A–I) Images of the subcellular location of BBR in cells before and after 0.5 µg/ml BBR administration for 1 h. (A–C) HepG2 cells; (D–F) HeLa cells; (G–I) SY5Y cells. The fluorescence of BBR is shown in green, differential interference contrast (DIC) figures represent the scale of cells and scale bar is 10 µm.

The intracellular distribution of BBR is important for understanding the role of BBR as an anti-cancer drug. We employed three representative cell lines: HepG2, HeLa and SY5Y to analyze BBR distribution [23]–[26]. A non-toxic dose of BBR (0.5 µg/ml) was used throughout the entire study based on the results of cytotoxicity assay and lethality curves (Figs. 1A–F). The confocal microscope depicted BBR distribution in living cells (Fig. 2A), revealing that BBR can enter the HepG2, HeLa and SY5Y cells within 1 h after drug treatment (Figs. 2B, C). The majority of BBR was in the cytoplasm and no obvious nuclear entry was observed. Previous reports have showed BBR enter nucleus and compete with TBP (TATA Box Binding protein) for TATA Box in PC12 cell [14]. However we failed to observe the nucleus distribution of BBR in these three cancer cell lines.

**Figure 3:**
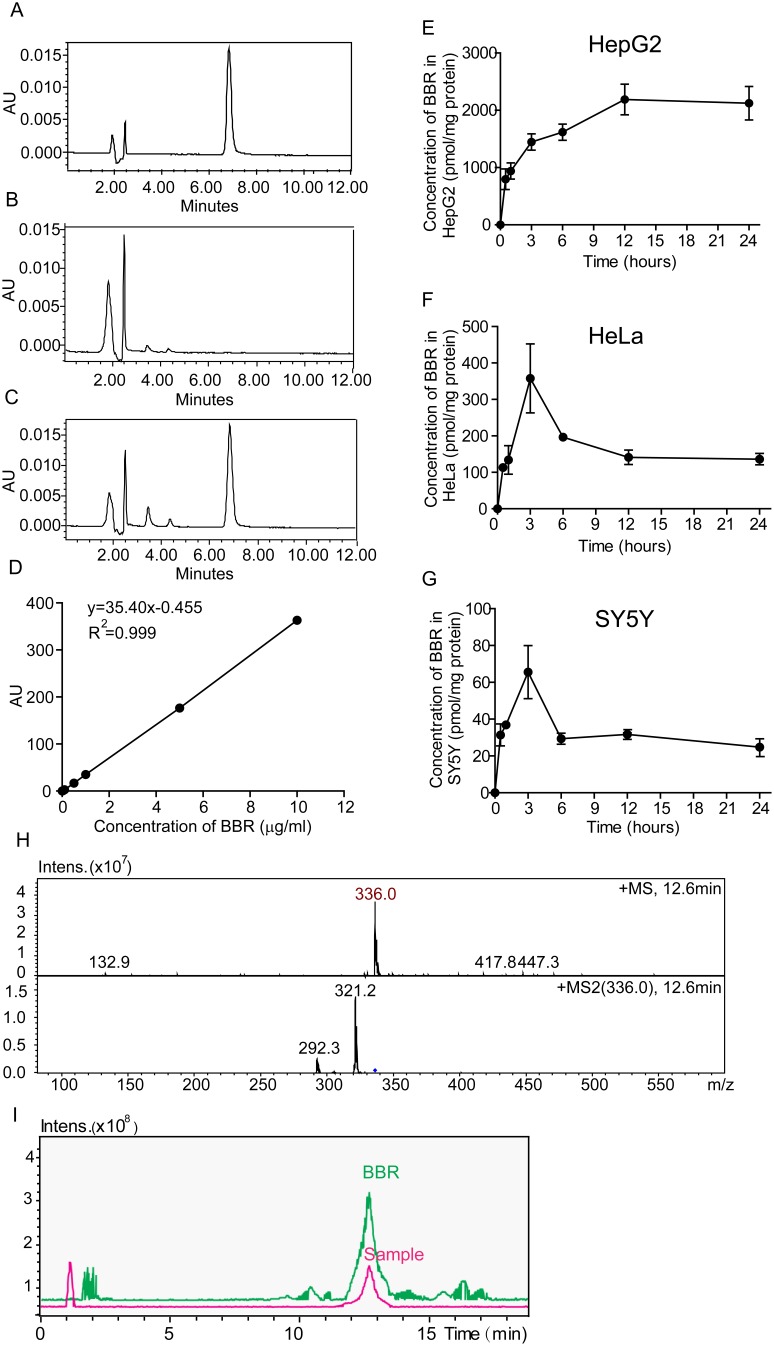
Chromatograms and kinetic behavior of BBR in the three cell lines. (A–C) HPLC chromatograms. (A) Standard BBR; (B) Blank sample from BBR treated cells; (C) Samples from cells with BBR administration for 12 h. (D) Standard curves of BBR concentration verse AU intensity using HPLC assay. (E–G) Kinetic behavior of BBR in the cells in plot with the administration time in 24 h. (E) HepG2; (F) HeLa; (G) SY5Y cells. (H, I) LC/MS chromatograms of BBR in HepG2 cells after the administration for 24 h. (H) the mass spectrum of BBR; (I) the chromatogram of BBR. Standard BBR was shown in green; the sample was shown in purple color. Data are presented as mean ± S.D. from six independent experiments (n = 6).

Then we used HPLC and LC/MS to quantitatively analyze the intracellular concentration of BBR in HepG2, HeLa and SY5Y cells after cellular entry. Results showed that BBR can be effectively separated and detected using HPLC under the indicated conditions (Figs. 3A–C). The standard curves of BBR demonstrated a strong signal-to-noise ratio and a good linear relationship (R^2^ = 0.999) between the peak area and the concentration of BBR (Fig. 3D). There were differences in the uptake of BBR among the three cell lines. HPLC assay showed that in HepG2 cells, BBR maximum concentration (C_max_) was at around 2188.8 pmol/mg at approximately 12 h after treatment (Fig. 3E). The C_max_ of BBR in HeLa and SY5Y were 357.8 pmol/mg and 65.5 pmol/mg, respectively (Figs. 3F, G). After three hours, the concentrations of BBR in HeLa and SY5Y cells came down. It is shown that BBR was accumulated to higher level in HepG2 cells than the others.

Furthermore, to understand whether the higher BBR concentration in HepG2 cells was due to the role of HepG2 in actively metabolizing BBR, we set up LC/MS to analyze the metabolite products of BBR in the cells. As reported, there are metabolite products of BBR after oral or injection uptake in rats [27]. However, we did not detect any of them in HepG2 cells (Figs. 3H, I), suggesting BBR remained intact in HepG2 cells [28].

### P-gp is a major efflux transporter of BBR

**Figure 4:**
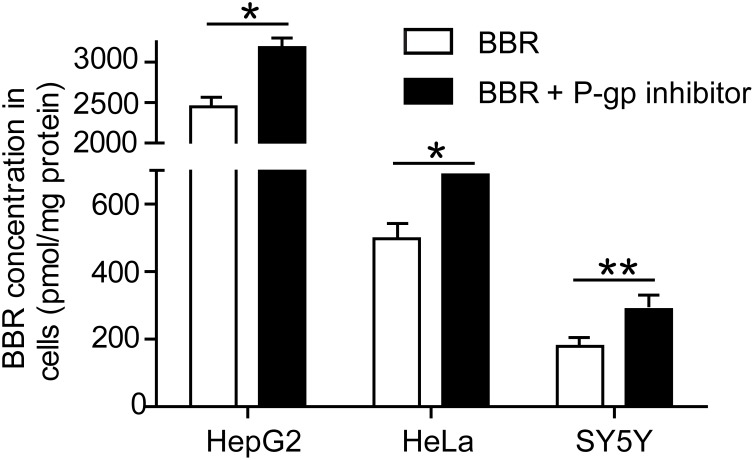
Effect of P-glycoprotein (P-gp) inhibitor on BBR uptake in HepG2, HeLa and SY5Y cells. Cells were treated with 0.5 µg/ml BBR and 0.3 µmol Zosuquidar, the inhibitor of P-gp activity, for 12 h. The intracellular concentrations of BBR were determined by HPLC assay. Data are presented as mean ± S.D from three independent experiments (n = 3). * *v.s.* the control, *P*<0.05. ** *v.s.* the control, *P*<0.005, *t*-test.

To validate the role of P-gp in the efflux of BBR in HepG2, HeLa and SY5Y cells, a selective P-gp inhibitor, Zosuquidar (LY335979, Selleckchem, USA) was employed [29], [30]. As shown in Fig. 4, Zosuquidar efficiently increased the concentration of BBR in HepG2, HeLa and SY5Y cells, suggesting that P-gp is a major BBR efflux transporter in these cells. These results were in good agreement with previous studies [28], [31].

### Molecular docking of BBR to P-gp

**Figure 5:**
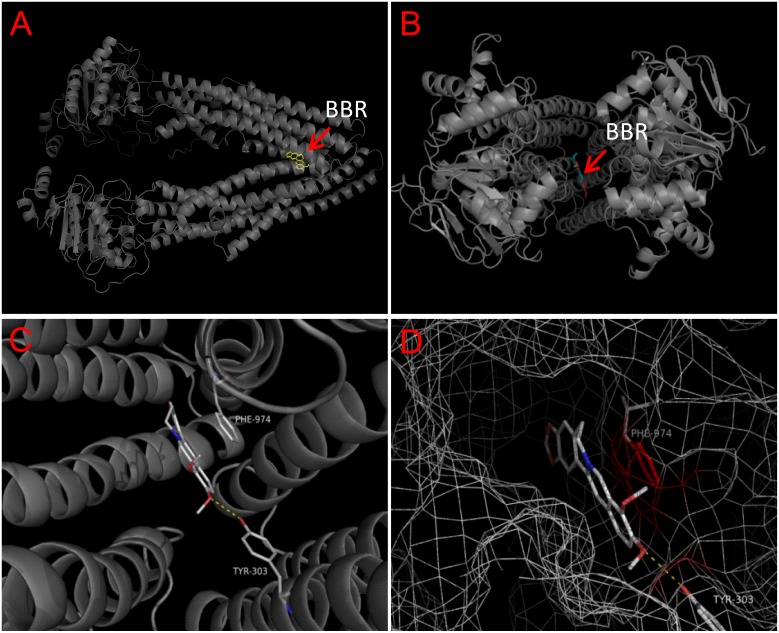
Molecular docking of berberine (BBR) to Homo sapiens P-gp. (A) Side and (B) top view of the BBR binding pocket of P-gp. (C, D) the overall docking views of BBR in the binding pocket of P-gp. Blue mesh: protein's electron isodensity map around the binding site. Protein are represented as cartoon (transparency = 20%), small molecules and important amino acids (as labeled) are represented as lines (white and green, carbon. red, oxygen. blue, nitrogen). Yellow dash line, hydrogen bond.

We further analyzed the interaction of P-gp and BBR by computer based molecular docking. The crystal structure of human P-gp has been identified from the database of NCBI. We therefore utilized the same structure as receptor to perform molecular docking of BBR. Results suggested that BBR was able to bind to the drug binding-pocket of P-gp (Figs. 5A, B). The binding site of BBR was located in the “lower” part of the binding pocket (Figs. 5C, D). The glide energy is −9.1 kcal/mol, suggesting the high binding affinity of BBR to P-gp.

### BBR affects P-gp expression

**Figure 6:**
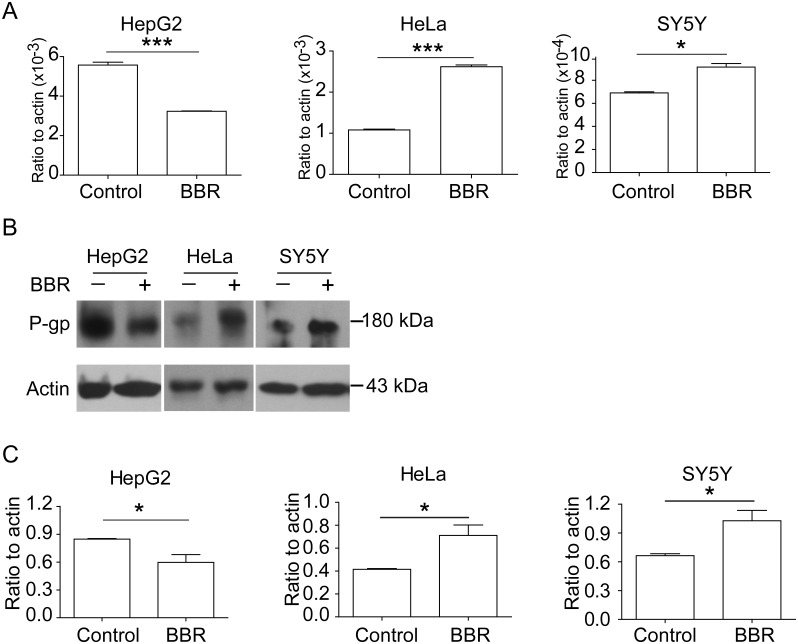
BBR modulates the transcription and expression of P-gp. (A) mRNA expressions of endogenous P-gp using real-time PCR assay. (B) Western blotting assay showed changes of P-gp protein with BBR treatment. (C) The ratio changes of P-gp versus β-actin. Data are presented as mean ± S.D. from 3 independent experiments (n = 3). The concentration of BBR was 0.5 µg/ml. * *v.s.* the control, *P*<0.05; ** *v.s.* the control, *P*<0.005, *t*-test. For panels in (A) and (C), Left, HepG2; Middle, HeLa; Right, SY5Y.

To elucidate whether BBR regulates P-gp expression, we examined the mRNA and protein expression levels of P-gp in HepG2, HeLa and SY5Y cells after BBR treatment. Results showed that the mRNA of P-gp was increased in HeLa and SY5Y cells, while decreased in HepG2 cells in a dose-dependent manner after BBR treatment (Fig. 6A). Furthermore, Western blot analysis validated these results, showing that BBR increased P-gp expression in HeLa and SY5Y cells, but decreased the expression in HepG2 cells (Figs. 6B, C).

**Figure 7:**
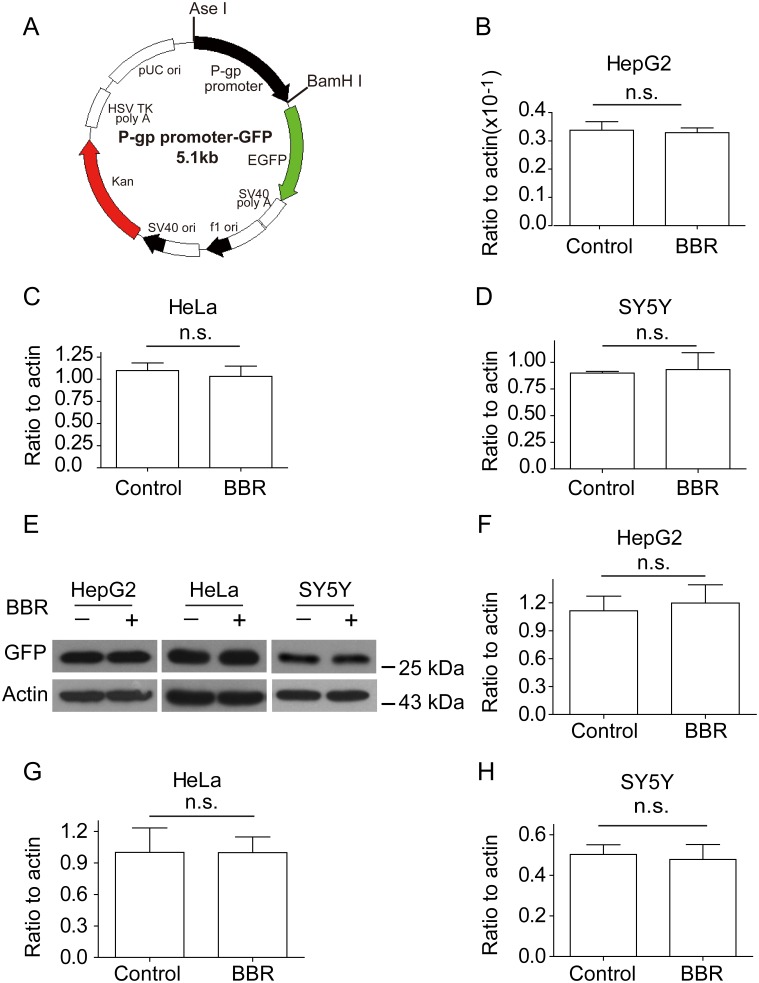
BBR does not act on P-gp promoter. (A) Schematic map of P-gp 1 kb promoter fused GFP (P-gp promoter-GFP) construct. Black, P-gp promoter. Green, reading frame of EGFP. (B–D) mRNA expressions of P-gp 1 kb promoter fused GFP in the presence or absence of BBR by real-time PCR assay. (B), HepG2; (C), HeLa; (D), SY5Y. (E) Western blotting assay showed changes of P-gp 1 kb promoter fused GFP protein with BBR treatment. (F–H) The ratio changes of GFP versus β-actin. Data are presented as mean ± S.D. from 3 independent experiments (n = 3). “*n.s.”* means no statistical significance.

How BBR regulates P-gp expression is unknown. To determine the effect of BBR on P-gp transcription initiation, a P-gp 1 kb promoter fused GFP was constructed (Fig. 7A) and transfected into the three cell lines [19]. These cells were treated with or without BBR and followed by flow cytometry selection and RT-PCR analysis (Figs. 7B–D). RT-PCR results revealed that BBR has no regulation role in the promoter activity of P-gp. Besides, the protein expression level of GFP was not affected by BBR according to the Western blotting results in Figs. 7E–H. Since GFP was fused with P-gp promoter in the plasma and it is not present in mammalian cells, GFP protein expression data demonstrated that BBR couldn’t act on P-gp promoter at the transcript level.

## Discussion

The purpose of this study is to understand the interaction between BBR, a potent anti-cancer compound, and P-gp, a widely distributed drug efflux transporter. Previous studies revealed that at a concentration of 0.5 µg/ml BBR was effective in protecting cultured neuron cells from damage after oxygen and glucose deprivation [22] and we verified it did not cause any damage to HepG2, HeLa or SY5Y cells based on MTT assay. In our present work, we observed the entrance of BBR by confocal microscope and recorded the dynamic accumulation of BBR using HPLC and LC/MS. Results showed significant differences among the three cell lines, HepG2 accumulated more BBR for longer duration than the other two cell lines. By LC/MS assay, we found there were no metabolites of BBR in HepG2 cells after BBR administration, indicating the high concentration of BBR in HepG2 cells is only BBR itself. Therefore, we concluded that HepG2 could accumulate more BBR and keep it for a longer time in the cells.

As the literature reported [15], P-gp is mainly responsible for the efflux of BBR. Therefore we employed a specific inhibitor of P-gp, which efficiently blocked the efflux of BBR and increased BBR concentration in HepG2 cells. A specific binding pocket of P-gp for BBR was revealed in the molecular docking prediction. This partially explained the site through which BBR could affect the ability of P-gp in mediating BBR efflux, though a detailed mechanism correlating these two phenomena (the binding of BBR and the efflux efficiency of P-gp) is still unknown.

By applying molecular biology and biochemistry methods, we measured the levels of mRNA and protein of P-gp in the cells after BBR treatment. Results showed that BBR induced the mRNA and protein expressions of P-gp in HeLa and SY5Y cells, and consequently promoted the efflux of BBR and reduced the concentration of intracellular BBR. These results might explain the observation that the intracellular concentration of BBR peaked and decreased very quickly in these cells. However, P-gp expression decreased with increasing concentrations of BBR in HepG2 cells, which was in good agreement with the fact that the intracellular concentration of BBR continuously built up in HepG2 cells.

As shown in Fig. 6 and Fig. 7, mRNA and protein expressions of P-gp in HeLa and SY5Y cells were up-regulated after BBR administration, while mRNA expression of P-gp in the promoter transfected in the cells were not up-regulated. To verify the result, we performed a supplementary experiment using GFP protein expression. The results showed GFP protein expressions were not up-regulated after BBR administration, supporting the mRNA expression data of P-gp in the promoter transfected in HepG2, HeLa and SY5Y cells. Since there is no GFP protein in mammal cells, GFP protein expression data strongly support that BBR didn’t act on the promoter of P-gp.

BBR was reported to increase the bioavailability of several compounds by inhibiting P-gp in Caco-2 intestinal cells [16]. It also promoted the expression of P-gp in multidrug resistance of cancer cells including oral (KB, OC2), gastric (SC-M1, NUGC-3) and colon (COLO 205, CT 26) cancer cells [32], [33], suggesting the complexity of the relations between BBR and P-gp in different cells.

Since BBR couldn’t enter the nuclei, it is not likely that BBR has any action on the DNA/Gene sequences, leading to no effect of BBR on P-gp promoter transcription. Combine with the different P-gp expression patterns induced by BBR, we predict that the effect of BBR on P-gp expression should occur after gene transcription, for example mRNA and protein stability, or protein posttranscriptional modification. Employment of P-gp inhibitor drastically inhibited P-gp’s function of transporting BBR in the three cell lines, indicating that there are no differences among the function of P-gp protein in the cells. This suggests that there may be different cellular factors for P-gp protein expression in the cells and the interplays among BBR, P-gp and various signal transduction pathways might be drastically different in different cancer cell lines, which results in the different response of tumor cells to BBR. This requires further study.

## Conclusions

In summary, this is the first report of how BBR regulated P-gp activities among HepG2, HeLa and SY5Y cancer cell lines. P-gp combines with BBR in order to discharge BBR, and simultaneously BBR interferes with P-gp expression to regulate its own efflux from cytoplasm. BBR induced the expression of P-gp in HeLa and SY5Y cells, but inhibited the expression of P-gp in HepG2 cells, probably caused by distinct patterns of P-gp protein translation. There was no direct regulation of P-gp promoter transcription, indicating differences in regulation or/and mechanisms that exist in HepG2 cells and HeLa and SY5Y cells. The differences among the three cell lines indicated in this study may help us understand cancer cells’ abilities to uptake and transport anti-cancer drugs, also aiding in the knowledge of possible drug resistance mechanisms.
